# The Behavioral Factors That Influence Person-Centered Social Care: A Literature Review and Conceptual Framework

**DOI:** 10.3390/ijerph19074334

**Published:** 2022-04-04

**Authors:** Eugene Tay, Ivo Vlaev, Sebastiano Massaro

**Affiliations:** 1 NUS Care Unit, Office of the Senior Deputy President and Provost, National University of Singapore, Singapore 119077, Singapore; 2 Department of Behavioural Science, Warwick Business School, University of Warwick, Coventry CV4 7AL, UK; ivo.vlaev@wbs.ac.uk; 3 Surrey Business School, University of Surrey, Guildford GU2 7XH, UK; sebastiano.massaro@theonelab.org

**Keywords:** elderly care, advance planning, decision-making, aging population, nudging

## Abstract

The last decade has seen numerous policy reforms to emplace person-centered social care. Consequently, the public has been given more information, choice, and autonomy to decide how best they want to be cared for later in life. Despite this, adults generally fail to plan or prepare effectively for their future care needs. Understanding the behavioral antecedents of person-centered decision-making is thus critical for addressing key gaps in the provision of quality social care. To this end, we conducted a literature review of the psychological and health sciences with the aim of identifying the aspects that influence person-centered decision-making in social care. Using an established theoretical framework, we distilled nine behavioral factors―knowledge, competency, health, goal clarity, time discounting, familiarity, cognitive biases, cognitive overload, and emotion―associated with “Capability,” “Opportunity,” “Motivation,” and “Behavior” that explained person-centered decision-making in social care. These factors exist to different degrees and change as a person ages, gradually impacting their ability to obtain the care they want. We discuss the role of carers and the promise of shared decision-making and conclude by advocating a shift from personal autonomy to one that is shared with carers in the delivery of quality social care.

## 1. Introduction

Over the last decade, person-centeredness, or an emphasis on providing care that is respectful and responsive to adults’ needs, has been at the forefront of many social care policy reforms in the United States (US), United Kingdom (UK), Canada, and many other countries (e.g., Germany, Australia). The US 2010 Affordable Care Act contains multiple provisions to increase healthcare value, quality, and efficiency through choice and information [1]. Likewise, the UK 2014 Care Act replaces a ‘one size fits all’ approach with a ‘person-centered’ health and social care system that focuses on addressing individuals’ needs and wellbeing [2]. These reforms generally seek to enhance individuals’ autonomy and responsibility in deciding how they want to be best cared for in the latter part of their lives, including the type of support they may need (e.g., transport, home adaptations, nursing staff), where they may want to receive care (e.g., home or hospice), and how they may want to fund them (e.g., insurance). Studies suggest that advanced care planning improves the receipt of person-centered care and increases satisfaction with care [3,4,5], and may even lower the cost of care [6].

Yet, people who develop care needs often fail to plan and decide on a range of matters earlier in their lives. Using data from the 2016 Empire State Poll conducted in New York, researchers established a poor level of awareness among participants in being able to define hospice care (83%) and palliative care (27%), and identified several common areas of misconceptions, such as associating hospice care with end-of-life care (60%) [7]. In the UK, a 2017 survey suggested that about 47 percent of the population wrongly believe that social care is free at the point of need, and only 35 percent had made financial plans for their future care [8]. Even though aging and age-related changes affect everyone, people tend to avoid the topic and only consider their potential care when the need arises. While some may claim to be more risk-averse and be avid planners, most will simply react in response to a certain event, such as a medical crisis or the passing of a close one [9]. This is when people are least likely to cope with choice, information, and stress to make a proper person-centered decision. Indeed, the current COVID-19 pandemic provides a timely demonstration of how fear and paranoia in people, brought on by sudden changes in an uncertain situation, can increase stigmatization of care professionals [10] or increase susceptibility to misinformation [11,12].

Person-centered decisions about social care are rarely clear-cut. They involve multiple factors and require considerable foresight about future needs. Thus, identifying and addressing the behavioral antecedents of person-centered decision-making in social care is a fundamental call for both public health and public policy. More insights on this will surface the behavioral barriers and facilitators of advance care planning and highlight areas in need of practical interventions to promote person-centered social care. In this work, we aim to address this scenario by performing a literature review on the landscape of elderly social care, with the goal of identifying which aspects and in what ways they can influence person-centered decision-making about older care needs. Given the impetus to promote person-centered decision-making in policy and practice in countries like the UK, US and Canada, this review will mainly draw insights from these societies to inform research on social care.

This paper contributes to knowledge in three main ways. Firstly, by adopting a behavioral lens, we aim to systematize and synthetize the crucial factors that influence decision-making about social care. In doing so, we incorporate insights from the behavioral sciences to address possible limitations that exist at the individual-policy and individual-practice interface. Secondly, we explore and reveal the importance of shared decision-making in social care, which until recently has been largely confined to the health and medical sciences. Thirdly, we present a novel framework for conceptualizing the behavioral factors and the conditions under which they may be more (or less) crucial in promoting person-centered social care. Altogether, this work provides a comprehensive toolkit for policymakers and practitioners to consider a wide range of decision-making factors that might influence the receipt of quality social care.

## 2. Methods

We performed a narrative review on the barriers and facilitators that affect person-centered decision-making for elderly care needs. We focus on societies like the UK, US and Canada where considerable headway has been made to emplace person-centered social care in policy and practice. We adopted the COM-B framework [13,14] for the purposes of guiding the identification and classification of key behavioral factors and conceptualizing their interrelationships. The COM-B framework recognizes ‘behavior’ as a system of multiple interacting components, comprising ‘capability,’ ‘opportunity,’ and ‘motivation’. The framework has been used extensively in healthcare to address unhealthy eating habits, poor physical activity, and cigarette smoking in the general population [13,15]. Using a theory-driven approach to behavior change allowed us to identify 10 key factors from the psychological and health sciences that could affect person-centered decision-making in predictable ways. We abridge the findings from the retrieved literature into four overarching components: (1) capability, (2) opportunity, (3) motivation, and (4) behavior. In the following section, we explain the evidence behind these factors and elaborate on how third parties, including government entities, formal carers, and informal carers play a role in promoting person-centered elderly care.

## 3. Results

### 3.1. Capability

Capability refers to a person’s decisional capacities to reason and deliberate, express preferences and values, understand one’s circumstances, comprehend given information, and communicate a choice [16]. Hence, this section examines factors that affect person-centered decision-making well before people lose the capacity to decide their own best interests.

#### 3.1.1. Knowledge

Knowledge about the ways in which the social care system operates is one of the greatest hurdles in getting people to consider their potential care needs. Using nationally representative data from the Health and Retirement Study (*n* = 1504), Bakk and Cadet [17] found significant differences in knowledge about the Medicare Low-Income Subsidy scheme, such that compared to older non-Hispanic Whites, older non-Hispanic blacks and Hispanics, as well as those with a Spanish-speaking preference, were much less likely to know about it. Some hints of poor public knowledge about social care are also evident in the 2018 British Social Attitudes (BSA) survey [18]. The BSA survey, which recruited a nationally representative sample of 2926 people between July and October 2018, asked about satisfaction with health and social care and found a relatively high proportion of ‘don’t knows’ and ‘neither satisfied not dissatisfied’ related to social care (9% and 31%, respectively) compared to GPs (<1% and 13%) and outpatient services (3% and 15%) [18]. This points to a poor level of public awareness regarding social care and that such deficiencies may be greater along racial and cultural lines. Crucially, poor awareness may hinder those with the greatest needs to benefit from any targeted policy.

Educational programs could be one effective means for promoting person-centered decision-making in social care. For example, Detering et al. [19] studied the effects of advanced end-of-life care planning by randomizing 309 legally competent medical inpatients, aged 80 and above, into either a control group (*n* = 155) that underwent usual care or an intervention group (*n* = 154) that received usual care plus information and support on advance planning. After six months, the researchers found that among the patients who had passed away, those belonging to the intervention group were much more likely to receive the end-of-life care that they had wanted than those who had been assigned to the control group. Alternatively, making advance planning seminars available to the public could help individuals understand the importance of preparing ahead of time. Studies show that individuals who attend financial preparation seminars tend to develop a more favorable attitude towards retirement, acquire more knowledge on the basics of financial planning, engage in financial preparation [20] and contribute more to their own retirement fund [21]. Knowledge about the social care system, including how it works, who funds it, and who provides it, is thus an important aspect in promoting person-centered care. Exposing people to these pieces of information, preferably at an earlier rather than later age, may not only reduce the awareness gap in social care, but also drive up commitment towards advanced planning, thereby increasing a person’s likelihood of getting the care they want.

#### 3.1.2. Competency

Current policies on social care stress the importance of informed decision-making in person-centered care. Yet, such a cognitive exercise usually requires a set of decision-making skills for people to effectively understand complex information, appreciate risks and uncertainties, weigh the tradeoffs involved, and make rational choices.

Literacy―the ability to read, write and understand information―and numeracy―the ability to comprehend and apply numerical concepts―are critical skills for making objective assessments [22,23,24]. In an examination of 1400 patients at a public health system in San Francisco, Nouri et al. [25], established a negative association linking health literacy to knowledge about advanced care planning. Likewise, a large survey of 784 adults aged 55 to 74 sampled from the Chicago area showed that individuals low in literacy were much less likely (about 0.45 times) than those with high literacy to possess an advance care directive [26]. These findings suggest that decision-making skills may have motivational properties in person-centered social care. Without intervention, adults with poorer decision-making competencies may be at greater risk of being ‘left behind’ by recent policy changes.

A competency gap can be addressed through tailored communication, including the use of non-technical terms, absolute numbers (3 of 1000 people) instead of percentages (0.3%), and graphical illustrations instead of text [27]. Such strategies have been shown to improve individuals’ understanding and satisfaction in complex care decisions, even for those with poorer decision-making skills [28]. This suggests that the way in which third parties share information may be an influential determinant of quality in person-centered care. Despite this, little is known about the current state of literacy and numeracy among those considering social care options, or if these competencies affect decision-making in different ways, like the desire for involvement, gains in knowledge, risk aversion, and health outcomes. More research is needed to elucidate the relationship between quality decision-making and quality social care.

### 3.2. Opportunity

Opportunity are factors beyond the direct control of the individual that can prompt or make a specific behavior possible. In social care, the opportunity to make a person-centered decision will usually rise and fall depending on the situation.

#### Health

Aging brings significant changes to a person’s health status and care needs. It affects sensory (e.g., vision, hearing, and smell) and physical functions (e.g., motor ability), both of which may develop differently depending on a person’s medical condition (e.g., diabetes, dementia) and lifestyle (e.g., exercise, diet, and tobacco and alcohol use). For these reasons, a person’s entry and journey through the social care system rarely unfolds in a predetermined manner. For some, the desire to make home adaptations and maintain independence may arise when people anticipate, fear, or suffer minor mobility issues [29]. Individuals in these situations are arguably in a good position to make person-centered decisions. They are legally competent to make their own choices and are in a relatively low-stress state.

For some others, planning begins when they feel or are told that they might be losing their decisional capacity (e.g., dementia) [29], or when they start to suffer major mobility issues that hinder instrumental daily activities, such as cooking, showering, and housekeeping [30]. According to some health experts [31], age-related changes can threaten a person’s dignity and ability to secure their own wellbeing and thus, motivate protective actions. For instance, a decline in mental functioning may increase a person’s dependence on others to learn, manage, and fulfill their personal care needs. An interview with family carers of older adults who had been hospitalized for mental health-related issues revealed that the carer’s involvement in decision-making, in which they gathered information, consulted preferences, and communicated with care professionals about treatment plans, helped ensure that older patients got the care they wanted [32].

Finally, there are people who react to unplanned emergencies (e.g., falls). Medical crises are among the most complex and challenging ways for people to enter the social care system in that they would have very limited time to consider all available information and choices, nor be in a calm and composed state to make a rational decision. In cases of mental incapacitation, individuals may not even get the opportunity to express their preferences beforehand and so the next-of-kin becomes their medical proxy or surrogate for enabling person-centered care [33]. According to one study relying on data from the National Health Interview Survey (2009–2011; *n* = 16,720), the risk of delayed care due to cost or lack of transportation was greatest for older people living alone or with unrelated others [34]. Age-related health decline appears to bring opportunities for others to collaborate and deliver responsive care.

Overall, the evidence suggests that deciding in good health gives people ample time to plan, prepare, and even prevent mishaps, whereas deciding in poor health generally compels one to satisfy their most immediate care needs with little real choice and freedom to get what they want. Deciding in good health increases one’s chances of attaining person-centered care.

### 3.3. Motivation

Motivation can be intrinsic (e.g., goals) or extrinsic (e.g., incentives), but is generally a process that energizes and directs behavior.

#### 3.3.1. Goal Clarity

Planning for a potential decline in health is a challenging and unpleasant process [30]. Such thoughts can trigger strong aversive feelings in people and put them off from considering future care needs in the first place [35]. In one investigation, Powell and colleagues [36] found that some older adults tended to regard minor home adaptations, like handrails and ramps, as attempts to medicalize their own homes. These physical installations become an indication of weakness and thus represent a threat to a person’s self-esteem and dignity. Because these attitudes and perceptions can be off-putting [37], having sufficient goal clarity to overcome these negative stereotypes is critical for people to consider their older care needs.

Individuals with a clear intention about what they want are more likely to overcome psychological barriers and take necessary actions to achieve those ends [38,39]. Research has shown that individuals with a clear goal, a future time perspective (i.e., preference for the long-term view as opposed to focusing on the past or present), and a long-term planning orientation tend to prepare for their future finances and contribute more towards their retirement [40,41]. For example, Jacobs-Lawson and Hershey [40] attempted to identify the psychological determinants of retirement planning in 270 middle-aged (M = 36.2) working Americans, stratified by geographical region. The researchers found that individuals’ knowledge of retirement planning, future time perspective, and financial risk tolerance influenced retirement saving behavior. These factors interacted with one another, which suggests that goal clarity may be one of several systems (e.g., knowledge and risk preferences) motivating early engagement with the care system.

#### 3.3.2. Time Discounting

An absence of cognitive load (i.e., stress and pressure) is generally more conducive for rational decision-making [42,43]. However, a lack of urgency can similarly demotivate people from advance planning. Researchers explain that people typically discount the value of future choices at different rates, depending on their age, level of self-control, and the cost of time spent waiting [44,45]. Hershfield [46] attributed this behavior to a lack of physiological connection to a person’s future self, in which he showed that experimental participants placed in a future state, through imagination or simulation, tended to allocate more of their current money to a savings account. While this points to intertemporal decision-making as being an individually motivated process, some recent works have begun to explore the influence of others on this, such as whether close ones help or hinder advance planning.

Delaying gratification, or the acceptance of small short-term costs in exchange for larger long-term gains, is difficult but predictive of many important life outcomes (e.g., retirement savings and physical wellbeing) [47,48]. Yet, delaying gratification also depends on individuals’ trust in people to deliver those future gains, especially in the context of managing older care needs. Michaelson et al. [49] investigated the causal role of social trust in delaying gratification and showed that participants (*n* = 250) were less likely to wait for delayed rewards from less reliable personas and that perceived trustworthiness predicted the extent to which participants delayed gratification. Furthermore, in Henning-Smith and Shippee’s [50] analysis of the 2012 National Health Interview Survey in the US, expectations about future care needs and support in adults varied significantly by their current living arrangement, such that those living with young children were least likely to expect any form of long-term care or support, while those living alone were most likely to expect some form of help in future. This hints that social trust, or the belief in others’ ability to meet personal older care needs, may be a critical dimension in motivating (and enabling) person-centered care.

#### 3.3.3. Familiarity

The performance of certain everyday tasks can become so routine and repetitive that they require little or no conscious effort to execute. Over time, individuals may develop a mental script on how to execute them (e.g., daily commute to the local community center), and mental schemas for categorizing, interpreting, and generalizing issues (e.g., residents from this neighborhood are nicer) [51]. Familiarity may explain why people prefer to stay comfortably at home and adjust (e.g., making home adaptations), rather than move into other types of sheltered care [52]. Efforts to promote familiarity in stay-home care are also evident in the Community Ageing in Place—Advancing Better Living for Elders (CAPABLE) program that is currently underway in the US [53]. The CAPABLE program addresses personal and environmental factors that contribute to disability by using a person-directed approach to help older adults manage pain, mental health, communication with health professionals, and other daily activities of their choice, whilst living in their homes. In a randomized trial comprising 300 low-income adults with a disability, the researchers showed that compared to the control group, those assigned to CAPABLE were more likely to report that the program helped them take care of themselves and increased their confidence in managing daily challenges [54].

On the other hand, individuals are likely to find the management of novel tasks, like navigating the social care system for the first time, especially complex and challenging. Studies show that older adults tend to find new technology (e.g., tablet device, video-conferencing) complicated to learn and use [55], even if it may be beneficial to their health and well-being, for example in battling social isolation during the COVID-19 pandemic [56]. Since learning requires controlled, deliberative processes [57,58], some individuals may shy away from difficult but essential matters (e.g., getting a mobility aid, teleconsultation) [59]. Hence, the expansion of telehealth and other tech-facilitated services must account for the different needs and capabilities of older adults. Otherwise, technology may exacerbate health disparities in underserved communities, rather than reducing them.

### 3.4. Behavior

Behavior is the amalgamation of capability, opportunity, and motivation to make social care decisions that are based on the best available information and centered on individuals’ preferences. However, even when individuals do decide to engage in decision-making, they normally possess limited attention span to process all available information in an objective manner [43,60,61]. The following summarizes some of the biases that can undermine rational decision-making in the context of person-centered care.

#### 3.4.1. Cognitive Biases

Individuals’ preferences are usually reference-dependent and averse to losses in that there is a greater motivation to avoid losses than to secure the same amount of gains [62]. As it applies to social care, people in good health may value future losses to illness and aging much more than those who are already in poor health, thereby predisposing the former to take precautionary measures, such as searching for information and getting health insurance [63]. Conversely, an optimism bias may cause people to underestimate the probability of an adverse event. For example, older adults may perceive their odds of tripping and needing emergency attention as much lower than what objective data may suggest otherwise. Such biases can affect objective assessments of care needs and hamper advance planning.

Furthermore, aging appears to trigger negative stereotypes in people [64]. Aging stereotypes in Western cultures are primarily negative, depicting later life as a period of ill health, loneliness, and mental and physical decline [64]. Auman and associates [65] investigated the relationship between aging-related stereotypes and anxiety and cardiovascular reactivity by using a mixture of self-reported and physiological measures. In a sample of 122 patients, randomized to either receive health-related primes (e.g., sickness, helplessness, dependence) or leisure-related primes (e.g., leisure activities), those in the health condition reported significantly higher levels of anxiety and blood pressure than those in the leisure condition. In explaining this phenomenon, Auman et al. [65], argued that aging triggers fears of frailty and illness, which discourages people from seeking medical attention before needs begin to develop. Similarly, Levy and colleagues [66] studied whether stereotypes of aging might affect decisions about when to die by recruiting a sample of 64 participants, evenly split between old adults (*n* = 32, M_age_ = 74 years) and young adults (*n* = 32, M_age_ = 25 years). They found that old adults exposed to negative stereotypes frequently declined life-prolonging procedures, while old adults exposed to positive stereotypes often accepted them. No such effect was observed in young adults. This suggests that stereotypes about aging may be particularly influential in the elderly.

#### 3.4.2. Cognitive Overload

Research has shown that excessive amounts of information can often overwhelm and deter people from having to decide at all [67]. This paradox, where excessive information paralyzes decision-making, is known as information overload [68]. Information overload may be particularly disruptive for individuals as they begin to age and lose cognitive capacity [69,70]. Older adults tend to consider fewer pieces of information before making a decision, pay more attention to positive material rather than negative material [71], and rely on simpler decision-making strategies that may lead to poorer outcomes (e.g., choosing less profitable stocks) [72]. A pure informational approach puts older adults in a vulnerable position and a reliance on third parties may be necessary to compensate for declines in mental functioning.

Likewise, having more to choose from is not always better. Research suggests that individuals encountering a large assortment of options are usually more ambivalent, less satisfied, and less likely to make any choice at all than those with a smaller choice set [73,74,75]—a phenomenon known as choice overload [75]. In the US, a roll-out of more than forty Medicare coverage plans reportedly overwhelmed people. Few senior US residents found such “choice” helpful and a majority (about 73%) felt that the plans were “difficult and confusing” to understand [76]. In a more telling experiment on the dangers of choice overload, Hanoch et al. [77] recruited 192 healthy participants from California, half aged 18 and older, and half aged 65 or older, and randomized assigned them to one of three conditions containing 3, 10 or 20 Medicare drug plans. The researchers found that old age and a bigger choice set were responsible for fewer correct answers (i.e., deciding on a plan that minimized total annual cost), which raises questions about the ability of older adults in navigating the wide variety of social care options available to them.

#### 3.4.3. Emotion

People are generally poor at predicting their ability to control visceral forces (e.g., anxiety, anger, hunger, pain) that may influence human behavior, especially when such feelings transcend “hot–cold” emotional states [78,79,80]. According to Loewenstein [81], people in a ‘cold’ state often fail to fully and accurately appreciate how ‘hot’ states might affect their behavior, whereas people in a ‘hot’ state tend to overestimate their ability to manage and control their current behavior. In social care, planning normally happens in a ‘cold’ calculated state. As a result, people may delay critical decisions (e.g., checking out nearby care homes) thinking that they can cope with crises as they come [30]. On the other hand, extreme discomfort (e.g., pain), accidents, and other emotionally-charged situations (e.g., fear, distress) can predispose people to make impulsive decisions [78,82,83]. Decisions under such circumstances are not only more likely to produce intense feelings of regret in people [84], but are also more likely to undermine quality person-centered care. Thus, finding ways to bridge the “hot–cold” empathy gap may be instrumental for promoting person-centered outcomes in social care. In times of crisis, having an additional decision-maker may help relieve some stress and encourage rational decision-making.

## 4. Discussion

Planning and informed decision-making can ensure individuals get the care they want, well before the onset of a health decline or crisis that impairs their ability to make person-centered decisions. Yet, getting people to seriously consider their future care needs is a complex and massive undertaking. It will require them to have the capability, opportunity, and motivation for considering such matters and even then, a host of cognitive and affective biases may undermine the process of decision-making. For these reasons, third parties such as informal carers (e.g., family, friends, neighbors) and formal carers (e.g., nursing professionals) play a pivotal role in ensuring the delivery of person-centered care.

### 4.1. Promise of Shared Decision-Making

**Figure 1:**
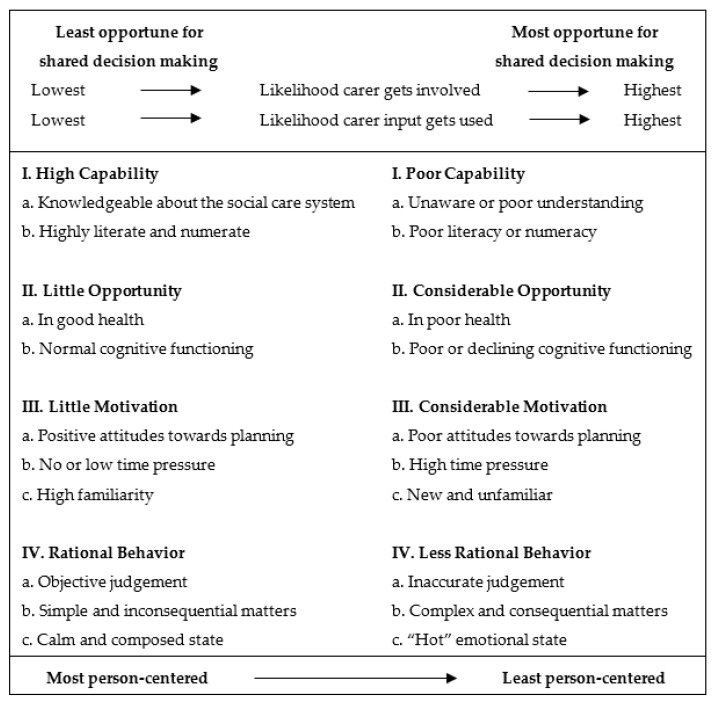
Impact model of shared decision-making in social care.

Shared decision-making is an attractive framework for enacting person-centered social care. Shared decision-making recognizes that carers bring precious informational, emotional, and relational value to an otherwise dull and stressful process. Figure 1 illustrates the ways and circumstances in which shared decision-making is most likely to improve the quality of person-centered social care.

The model conceptualizes the potential benefit of shared decision-making as one that is person and situation-specific. A person’s capability, opportunity, motivation, and behavior affect the extent to which shared decision-making functions as a crucial mechanism for enabling person-centered outcomes. In addition, the model points to some inherent tensions in the social care system. Conditions that are most attractive for quality person-centered social care (e.g., highly literate, highly familiar) are also the very ones that render shared decision-making less attractive. Conversely, conditions that are least ideal for quality person-centered social care, such as experiencing age-related declines in mental functioning, make shared decision-making ever more important in delivering responsive care. Therefore, how rapidly people shift from one extreme to the other is an important factor in social care.

The impact model of shared decision-making recognizes person-centered social care as a complex and dynamic process involving multiple factors and agents. More importantly, it illustrates how shared decision-making with carers may help older adults get the care they want. One prime example showcasing the benefit of carers can be found in Braun and colleagues’ [85] randomized controlled trial, which compared the effects of employing care managers to help Medicare beneficiaries navigate decision-making about cancer screening against standard cancer education. They showed that by sharing the process with Medicare beneficiaries, care managers were able to significantly increase various cancer screening uptake (e.g., breast cancer, prostate cancer), even in groups with significant disparities (e.g., Hawaiian, Filipino, Japanese) [85]. Likewise, informal carers are usually close acquaintances who live in close proximity or share a close relationship with the older adult they care for. As a result, informal carers tend to have shared lived experience, shared understanding of the situation, and shared expectations of the future, which make them highly knowledgeable about the needs and preferences of those whom they care for [86]. This puts informal carers in a good position to enable person-centered social care as they can help older adults meet physical and relational needs directly, and serve as surrogate decision-makers in end-of-life care [87].

Overall, carers are a crucial and increasingly fundamental component of person-centered social care. They are not only information agents (e.g., nudge advance planning, help with information search), but can also contribute directly to a person’s wellbeing (e.g., assist with commutes to a hospital).

### 4.2. Theoretical Implications

Existing social care policies that promote choice and information in the system overlook the barriers of engaging in the topic itself, much less the difficulty of navigating through the myriad of care options, each with its own costs, benefits, and potential to change over time. Efforts to promote quality social care should be both broad in tackling the various behavioral factors as well as tailored in adapting interventions to suit the needs of specific population groups. In so far as the individual is cognitively capable, these nudges to support decision-making should improve the odds of obtaining care that is aligned to their needs and preferences. On the flip side, carers are set to play a starring role in quality social care as individuals begin to suffer losses in cognitive, sensory, and motor functioning. Figure 1 is the first conceptual model to indicate the areas and degrees of responsibility wherein a shared relationship, as opposed to an autonomous one, might be more beneficial for person-centered social care. Therefore, the ability of carers―both formal and informal―is another important quality dimension in social care.

In addition, several factors allude to the role of perceived psychological safety in motivating decision-making about social care. Health deterioration, a distrust in family members, and a poor perception of nursing homes may threaten a person’s perceived sense of safety and motivate preparation and planning. Relatedly, emotional regulation may be another important factor in modulating a person’s response to threats concerning their wellbeing. Many issues in social care can trigger negative and aversive feelings in people. Studies suggest that people who can regulate their emotions effectively often make more accurate risk perceptions [88], are more resourceful in coping with challenges [89], and are more likely to voice problems [90]. Consequently, people with this ability stand to gain the most from person-centered social care; they are not only better equipped to make objective evaluations but are also more capable of doing so in times of uncertainty and duress. A person’s ability to regulate their own emotion may be a crucial factor in person-centered decision-making about social care.

### 4.3. Practical Implications

This review has shown that decision-making is susceptible to a range of behavioral influences. At the same time, it has also highlighted the value of effective communication in promoting person-centered decision-making. One such avenue would be to improve the science behind information and choice presentation to lay individuals. A review, commissioned by the Agency for Healthcare Research and Quality Effective Health Care Program in the US, examined the use of decision aids for adult advance care planning from the years 1990 to 2014 [91]. The review found that while most studies reported improvements in individuals’ clarity of preferences, decision knowledge, and decision confidence, the decision aids in these interventions tended to differ in terms of format, layout, interactivity as well as accessibility. Given the demonstrable benefits of decision aids, social care providers could leverage modern technology to make information more accessible, comprehensible, and customizable. This would allow people to manage their own cognitive load which could improve the quality of decision-making.

Furthermore, governments and organizations could regulate the structure and content (e.g., facilities, quality, and costs) in decision aids. Using a clear, standardized format may promote person-centered decision-making in people. Samanez-Larkin, Wagner, and Knutson [92] studied the impact of aging on financial decision-making by using neuroimaging techniques. The researchers found that using a simplified format to present critical information reduced the effects of distracting information on participants’ decision quality (i.e., optimal financial risk-taking), even for those older in age. Information transparency and standardization are thus key dimensions in person-centered social care and more must be done to foster such practices in existing providers.

Another possibility for promoting person-centered social care is to create dedicated services that can help older adults navigate the system. In the UK, several local authorities have developed ‘single points of access’, which are teams of specialists who are familiar with the resources in the region and are able to assess and match older adults to the most appropriate service, based on their needs and preferences [93]. Another initiative can be found in the form of social prescribing, in which general practitioners and other frontline staff have begun to recommend eligible patients to community-based services instead of offering only medicalized options [94]. These are just some examples of how social care organizations can help people overcome challenges associated with unfamiliar situations. More importantly, they lend support to the growing role formal and informal carers play in delivering social care.

### 4.4. Limitations

There are some limitations to this paper. Firstly, this literature review is not based on a systematic search of any specific literature, which makes replication and updating difficult. To mitigate a possible loss of relevant evidence, this paper relied on an established COM-B framework in the psychological and health sciences to structure the search. Moreover, the use of a theory-driven framework allows future researchers to extend beyond the themes identified in this paper. Secondly, as noted in the review, psychological aspects in decision-making are only one part of the equation necessary for promoting person-centered social care. The other concerns the access, availability, costs, and quality of social care services as well as their coordination in providing high-quality care. Whether psychological factors or supply-side factors are more responsible for health and care outcomes in older adults remains an open question, thus demanding further investigation. Thirdly, there is limited understanding on which COM-B factor most predicts quality social care. While a proper identification could facilitate policy analysis and refinement of interventions, more randomized controlled trials are needed to advance the science of person-centered social care.

## 5. Conclusions

The public is expected to play an increasingly active role in their own social care. In enabling the general population to do so, considerable emphasis has been placed on giving people more information, more choice, and more autonomy in deciding how they want to be cared for. This implicitly assumes that people know what they want—that they have clear, coherent preferences. It also assumes that people can do what they want—that they have complete focus and willpower to follow through with action.

However, as this review found, person-centered decision-making in social care is complex and difficult. Individuals are often unsure of what they want and even if they do, most people will find that they lack the necessary attention to evaluate all the available information and options in a careful manner. The aversive nature of social care normally deters planning and decision-making and as a result, decisions tend to be reactive, rather than the result of careful forethought.

Shared decision-making is a promising framework for addressing these issues and promoting person-centered social care. This review has shown how gaps in a person’s capability, opportunity, motivation, and behavior could be addressed with the involvement of others in decision-making. In this respect, governments, local authorities, service providers, and informal carers all form an invaluable part of a person’s ‘social care organization.’ They offer not just informational aid, but also relational and functional support in overcoming the various behavioral barriers that impede person-centered social care. For people to get the care they want, more emphasis should be placed on how decision-making unfolds, which usually necessitates (or could at least benefit from) the involvement of others. Only by appreciating the behavioral factors involved can quality person-centered social care be achieved.

## Data Availability

Not applicable.
